# Local ancestry inference provides insight into Tilapia breeding programmes

**DOI:** 10.1038/s41598-020-75744-9

**Published:** 2020-10-29

**Authors:** Alex Avallone, Kerry L. Bartie, Sarah-Louise C. Selly, Khanam Taslima, Antonio Campos Mendoza, Michaël Bekaert

**Affiliations:** 1 Institute of Aquaculture, Faculty of Natural Sciences, University of Stirling, Stirling, FK9 4LA Scotland, UK; 2 Department of Fisheries Biology and Genetics, Bangladesh Agricultural University, Mymensingh, 2202 Bangladesh; 3 Faculty of Biology, Universidad Michoacana de San Nicolás de Hidalgo, 58040 Morelia, Michoacán Mexico

**Keywords:** Bioinformatics, Population genetics, Animal breeding, Ichthyology, Genome informatics, Phylogenetics

## Abstract

Tilapia is one of the most commercially valuable species in aquaculture with over 5 million tonnes of Nile tilapia, *Oreochromis niloticus*, produced worldwide every year. It has become increasingly important to keep track of the inheritance of the selected traits under continuous improvement (e.g. growth rate, size at maturity or genetic gender), as selective breeding has also resulted in genes that can hitchhike as part of the process. The goal of this study was to generate a Local Ancestry Interence workflow that harnessed existing tilapia genotyping-by-sequencing studies, such as Double Digest RAD-seq derived Single-Nucleotide Polymorphism markers. We developed a workflow and implemented a suite of tools to resolve the local ancestry of each chromosomal locus based on reference panels of tilapia species of known origin. We used tilapia species, wild populations and breeding programmes to validate our methods. The precision of the pipeline was evaluated on the basis of its ability to identify the genetic makeup of samples of known ancestry. The easy and inexpensive application of local ancestry inference in breeding programmes will facilitate the monitoring of the genetic profile of individuals of interest, the tracking of the movement of genes from parents to offspring and the detection of hybrids and their origin.

## Introduction

Despite their prominent role in aquaculture, the volume of research involving tilapia is relatively low when compared to other fish species, like salmonids. As a result, existing research on ancestry tracing in tilapia is not abundant either. A fast and accurate method for tracing the hybridisation of admixed fish, i.e. fish of mixed ancestry, would uncover their composition, thus reconstructing their origins and even aiding identification of escapees and monitor introgression of native species. It would also help follow the movement of unwanted or unexpected traits alongside selected ones in a population, thus yielding useful information to produce more economically and environmentally favourable variants. Local Ancestry Inference (LAI) applications are more frequent in studies of dog breeds, as in the case of Alaskan sled dogs^1^, in which tracing of ancestry in sprint and long-distance sled dogs contributed to the identification of the genomic regions that correlated with performance-enhancing traits. Similarly, in humans, such tools have been more widely used to analyse how past migration events have impacted existing populations^2^ and to improve identification of ancestry-specific genetic susceptibility to disease in genome-wide association studies^3^.

Due to the relative scarcity of research specific to tilapia, or even fish in general, most of the literature currently available on inference of local ancestry focuses on human applications^4^. In over 15 years, more than 20 new LAI methods for human applications have been introduced^5^. Less often, relevant literature can be found on other animals like insects^6^ or, as already mentioned, dogs^7^.

While it is still possible to apply processes and tools developed for other animals to tilapia, a major obstacle persists, which is the vast difference in the amount, quality and variety of genotyped individuals available to build a reference panel. In humans, genetic studies often benefit from thousands^8^, if not tens of thousands, of individuals of certain descent, as well as publicly available data like that produced by the 1000 genomes project^9^. In tilapia, only hundreds of individuals are usually available, and it is much more difficult to accurately trace specific families, which limits the variety of the reference samples and negatively impacts the accuracy of phasing and LAI tools.

The selective breeding of tilapia revolves around the creation and maintenance of variants which would ideally display the most economically and environmentally favourable traits of their ancestors. Tilapia, and in particular Nile tilapia (*Oreochromis niloticus*), are highly common among breeding programmes due to their relatively short reproduction cycle, hardiness, and resistance to disease and parasites^10^. It has become increasingly necessary to track the inheritance of selected traits under continuous improvement, as selective breeding may also result in genes to hitchhike along in the process. Implementation of LAI in breeding programmes allows the monitoring of the genetic makeup architecture of each individual, the tracking the genes inheritance from parents to offspring, and this ensures that only loci of interest are selected by the breeding programmes.

The goal of this project was to generate a LAI workflow that harnessed existing tilapia genotyping-by-sequencing studies^11–13^, such as Double Digest RAD-seq (ddRAD) derived Single-Nucleotide Polymorphism (SNP) markers^14,15^. This provided an insight into breeding programmes with a more in-depth look at the genetic makeup of admixed individuals, significantly contributing to the identification of hybrids, and the development of new variants for aquaculture. We resolved the local ancestry of admixed individuals successfully and in detail, and the workflow was applied to the samples sourced from breeding programmes. We implemented a fast and accurate pipeline providing useful insights for breeding programmes of both tilapia and other animals, whether these are aimed at maintaining specific broodstocks or producing new variants.

## Results

### ddRAD library sequencing

High throughput sequencing of the animal from the four breeding programmes and additional individuals (93 individuals, Supplementary Table S1 online) produced 34,091,027 paired-end reads in total. After the filtering the reads, 82.5% of the total reads were retained (28,113,599 paired-end reads). The new reads as well as the published reads (275 samples; Supplementary Table S2 online) were mapped against the *O. niloticus* genome assembly (NCBI Assembly accession GCA_001858045.3). A total of 19,041 bi-allelic SNPs was extracted with a minor allele frequency (MAF) of at least 0.01, no deviation from the expected Mendelian segregation (*P* > 0.01) and common to at least 4 populations and 50% of their individuals (Table 1 and Supplementary Data S3 online).

**Table 1:** Tilapia species and populations.

Species	Location	Origin	Size	Abbrev.	Referenecs
*O. andersonii*	Zambia	Reference species	6	AND	Syaifudin et al.^11^
*O. aureus*	Israel	Reference species	10	AUR-I	Syaifudin et al.^11^
	Lake Manzala, Egypt	Reference species	5	AUR-E	Syaifudin et al.^11^
*O. karongae*	Lake Malawi	Reference species	4	KAR	Syaifudin et al.^11^
*O. macrochir*	Zambia	Reference species	4	MAC	Syaifudin et al.^11^
*O. mossambicus*	South Africa	Reference species	13	MOS-A	Syaifudin et al.^11^ and This study
	Zimbabwe	Reference species	9	MOS-Z	Syaifudin et al.^11^ and This study
*O. n. cancellatus*	Ethiopia, Lake Hora	Reference species	14	CAN-H	Syaifudin et al.^11^
	Ethiopia, Lake Hora	Reference species	11	CAN-K	Syaifudin et al.^11^
*O. n. filoa*	Ethiopia, Lake Metahara	Reference species	8	CAN-M	Syaifudin et al.^11^
*O. niloticus*	Ghana, Lake Volta, Kpandu	Reference species	12	NIL-K	Syaifudin et al.^11^
	Ghana, Lake Volta, Nyinuto	Reference species	12	NIL-N	Syaifudin et al.^11^
	Lake Manzala, Egypt	Reference species	22	NIL-E	Syaifudin et al.^11^ and This study
*O. u. hornorum*	Tanzania	Reference species	5	HOR	Syaifudin et al.^11^
*S. galilaeus*	Israel	Reference species	5	GAL	Syaifudin et al.^11^
*S. melanotheron*	Ghana	Reference species	4	MEL	Syaifudin et al.^11^
*T. zillii*	Ghana	Reference species	5	ZIL-G	Syaifudin et al.^11^
	Lake Manzala, Egypt	Reference species	5	ZIL-E	Syaifudin et al.^11^
Breeding programme	Colima, Mexico	Breeding programme	17	BRE-C	This study
	Morelos, Mexico	Breeding programme	18	BRE-M	This study
	Veracruz, Mexico	Breeding programme	18	BRE-V	This study
	Mexico	Breeding programme	18	BRE-L	This study
	Malaysia	GIFT programme	50	GIFT	Taslima et al.^13^

Species, geographical location, origin, size and abbreviation of the 23 sample populations.

### Population structures

**Figure 1:**
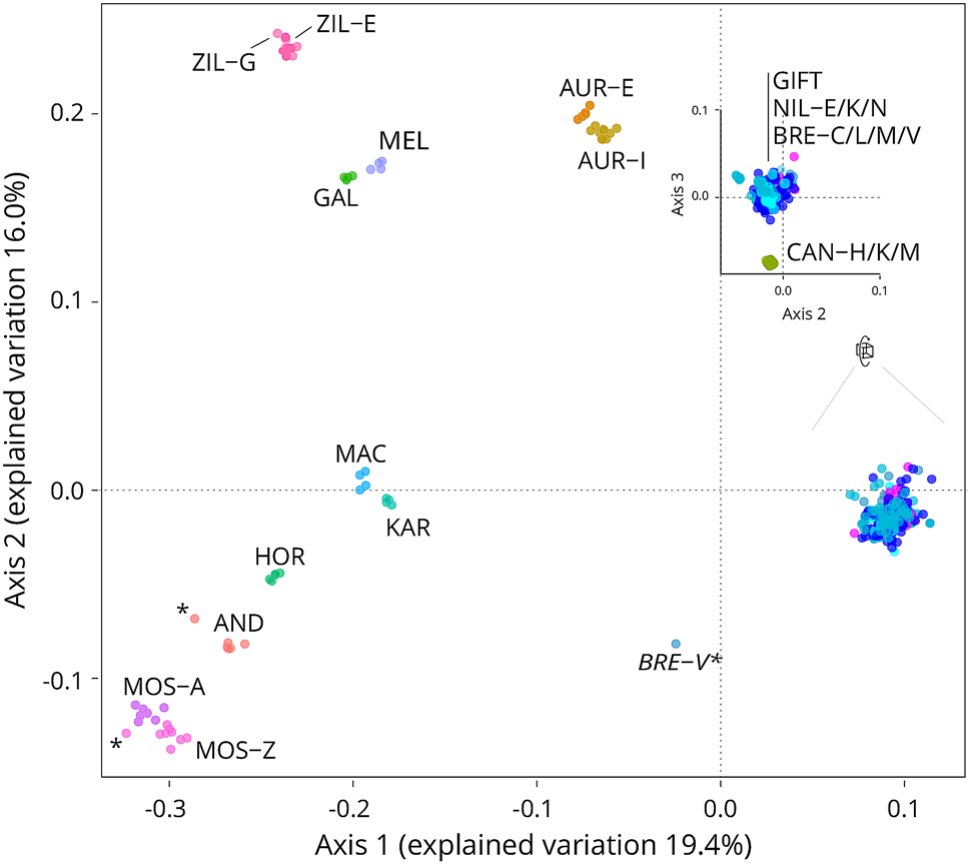
Multidimensional scaling analysis of identity by state results of the full dataset. First, second and third component explained 19.4%, 16.0% and 7.8% of the variation found. The inset on the top right, project the result on the second and third axes in order to distinguish the position of CAN-H/K and M compared to the NIL population and related populations (GIFT and BRE-C/L/M and V). Abbreviations included as listed in Table 1. *Indicate samples suspected to be hybrids (MOS-Z and AND) or outlier (BRE-V).

A Multidimensional scaling (MDS) analysis of identity by state (IBS) was utilised to separate the individuals into clusters based on their genetic distance^16^. This process grouped individuals of same origin together, while positioning the hybrids between the populations which more heavily contributed to their genome (Fig. 1).

The *O. n. filoa* (CAN-M) individual originating from Lake Metahara^17^ was grouped with *O. n. cancellatus* (CAN-H/K). As their similarity has prompted a proposition for a re-classification of *O. n. cancellatus* as *O. cancellatus*, with two sub-populations, *O. c. cancellatus* and *O. c. filoa*^18^, these samples were grouped with the remaining *O. n. cancellatus* populations due to the species being virtually indistinguishable.

Most populations were clearly resolved (Fig. 1, Table 1 for abbreviations), with the exception of the breeding programmes populations (BRE-C/L/M/V and Genetically Improved Farmed Tilapia (GIFT) programmes), which remained tightly clustered together with NIL-E/K/N (*O. niloticus* populations, their species of expected origin). For most populations, species-specific grouping was representative of the genetic closeness of their samples, e.g. AUR-E and AUR-I (*O. aureus*), or the three CAN (*O. n. cancellatus*) sub-populations: while still distinguishable, these populations of different origin were clustered under one (species) group. The same could be said for *O. niloticus*: the NIL-E, NIL-K and NIL-N sub-populations were grouped under NIL. Multidimensional Scaling Analysis also highlighted the presence of outliers, especially in the form of one MOZ-Z (*O. mossambicus*, Zambia), one AND (*O. andersonii*) and one BRE-V individual (Fig. 1, marked by a *). BRE-V disparate positioning was found to be due to the high incidence of missing genotypes in some individuals, rather than due to sample impurity. Finally, the genetic closeness of some species was noted, especially of some of those only represented by a single small population (KAR and MAC, or GAL and MEL), and was expected to cause ambiguities when trying to resolve the ancestry of individual samples.

### Ancestry inference

Before undergoing ancestry inference, these genotypic data were phased with BEAGLE^19^. Phasing is required to improve ancestry recognition, as separating the paternal and maternal contributions allows to infer the origin of each separately, since they could belong to different species or populations. Once these genotypic data are phased, RFMix separates each chromosome into a series of equally-sized windows, and the likelihood of each window belonging to each of the reference populations is calculated^20^. For each one, a random forest is trained to recognise the ancestry based on the reference panel: each tree of the random forest infers a putative ancestry, and a sum of all the *votes* determines the probabilities of that window originating from each possible ancestral population.

**Figure 2:**
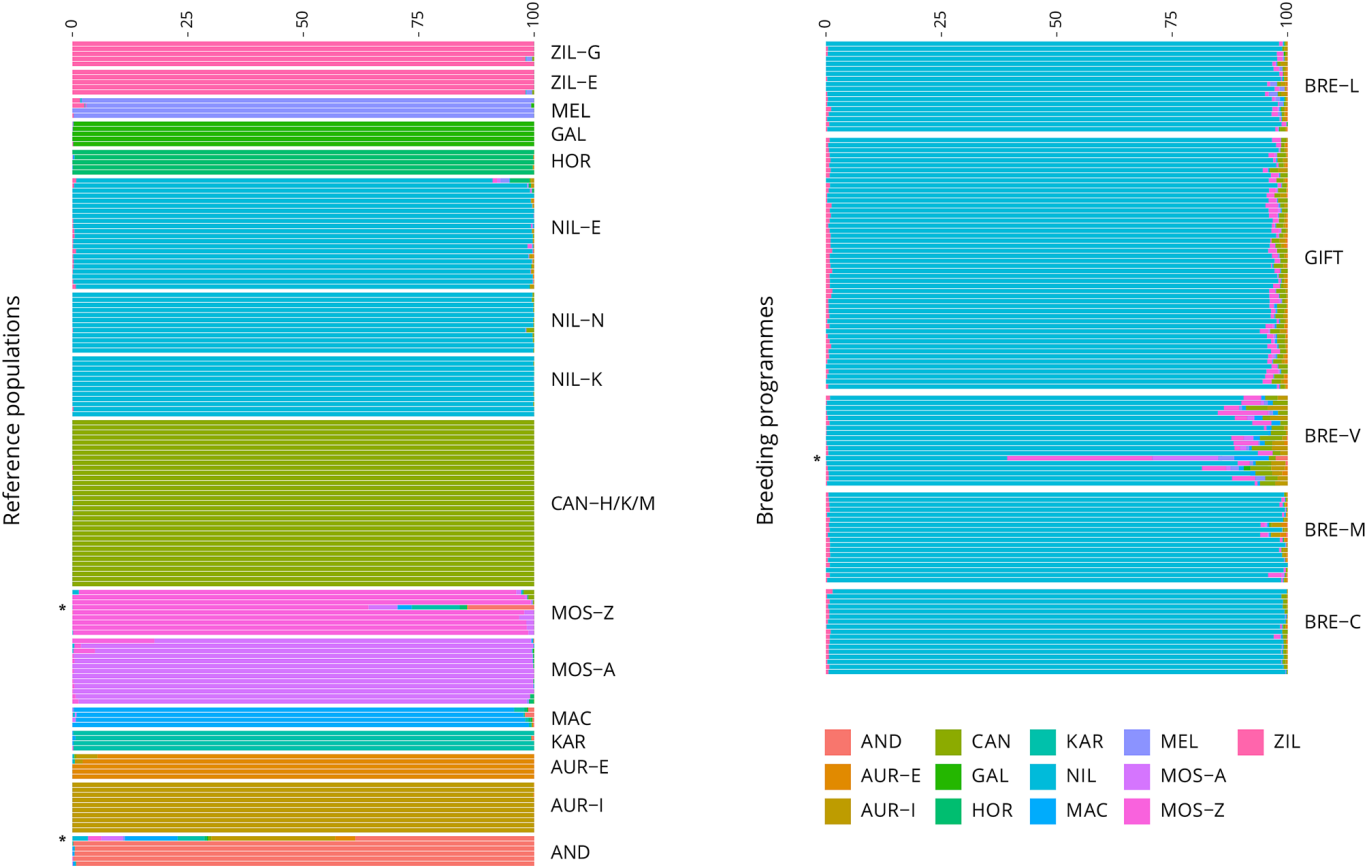
Global ancestry contribution. For each sample the predicted global ancestry contribution is reported. The reference sample global ancestry contributions were assigned based on a training set not including the breeding programme samples. * Indicate samples suspected to be hybrids (MOS-Z and AND) or outlier on the Multidimensional Scaling Analysis (BRE-V). Abbreviations included as listed in Table 1.

The inference accuracy using only the 155 references samples from 10 species was optimised to minimise the fragmentation, while maximising recognition of the reference samples (Fig. 2). The final combination featured 500 BEAGLE iterations, combined with 50 EM iterations and a SNPs window size of 7.

### Digital chromosome painting

**Figure 3:**
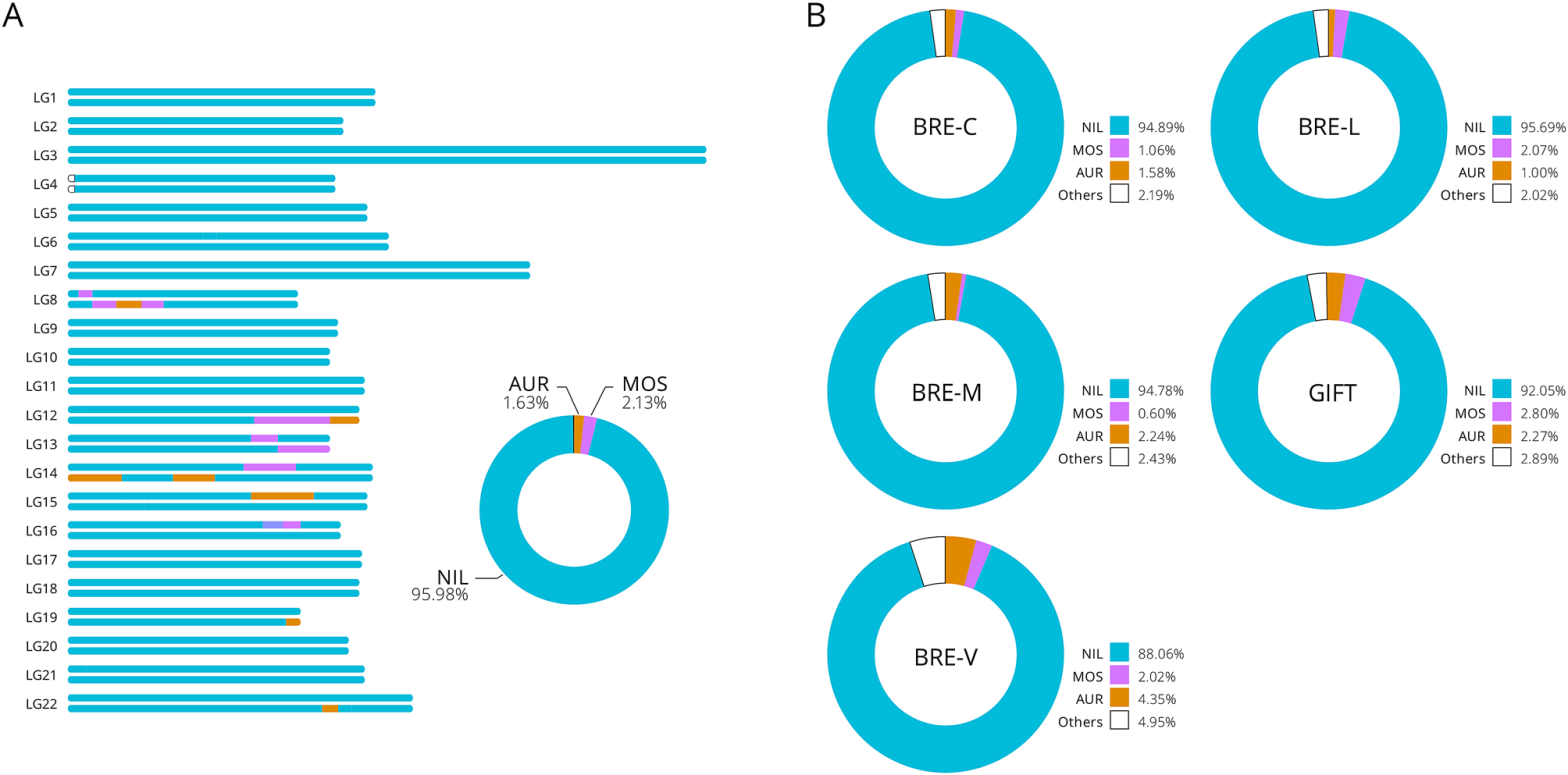
Local and Global ancestry of breeding programmes. (**A**) Local ancestry karyograms for the individual GIFT_12 (GIFT Breeding programme) with ancestry proportions similar to the estimated population averages. The *O. aureus* (AUR), *O. mossambicus* (MOS) and *O. niloticus* (NIL) haploid genomes present a complex mosaic of ancestry tracts across the genome, reflecting its demographic history; (**B**) Median ancestry proportions for breeding programmes, based on fractions of the chromosome length. Abbreviations included as listed in Table 1. Other, includes a low proportion of introgression that is more likely to be a consequence of a prediction ambiguity and noise rather than real introgression.

Using the reference samples as a training set, the breeding programme individuals were analysed for LAI. In contrast to the reference population, all of the individual samples exhibited a relatively high level of fragmentation (5 to 30%). As expected, the main contributor of the genome composition was *O. niloticus*, with variable contribution of *O. aureus* and *O. mossambicus* (Fig. 3). Several individuals showed a different composition (Fig. 2, samples marked with *).

## Discussion

Regardless of the species, LAI studies follow similar steps. First, a large number of markers are gathered from populations of known descent to build a reference panel^21^. The genotypes then undergo phasing to reverse crossing-overs, separating the contributions of the two parents^22^. The reference panel is then used to train the LAI model^21,23^. This also includes a “smoothing” algorithm, which improve the results by solving phasing errors, in case the maternal and paternal contributions have been swapped at certain loci, as well as genotyping errors, i.e. mistakes in the genome sequencing process that can be identified by their dissimilarity with the rest of the genome. Finally, the results are displayed graphically, on the chromosome^3^, or by probability representations^24^.

Intergenetic tilapia hybrids such as *Sarotherodon melanotheron* × *O. niloticus* as not uncommon and have been used in aquaculture to produce highly saline-tolerant hybrid^25^. Closer species crosses have commonly observed in the wild or feral populations. The ability of the methodology to correctly identify ancestries remains directly dependent on the “purity” and closeness of the reference samples, and on the quality of their genotyping: in fact, some species in this study could not be accurately recognised due to a high incidence of missing genotypes found in their samples (e.g. AND), the small sample number available (e.g. GAL or MEL), or because of their genetic closeness with other populations of small numbers (e.g. KAR and MAC), which prevented the algorithm from distinguishing the species appropriately.

While missing genotypes can be easily detected by examining the dataset, the same cannot be said for a lack of purity of the samples. Once the individuals are sampled and only sequenced SNPs remain from them, it is difficult to verify whether samples truly belong to the species and population they may be claimed to or if they have been mislabelled. Therefore, some of the uncertainty in the re-assignment of known ancestries may derive from unknown levels of admixture in supposedly pure samples. However, the ddRAD markers did support purity of species by tight species clustering.

*O. n. cancellatus*, also known as *Tilapia cancellata*, has been assimilated to *O. niloticus* since the sub-species was identified^26^, and is considered to be synonymous. The same is true for *O. n. filoa*. In this study these sample grouped with *O. n. cancellatus*. The results produced by this project, however, draw a clear distinction between NIL and CAN, as the multidimensional scaling analysis indicates that although genetically close, the groups do not overlap (Fig. 1). If samples of *Tilapia cancellata* were considered to be part of *O. niloticus*, and the two were mixed to form a supposedly pure broodstock, *O.  niloticus* would feature not only NIL, but also CAN markers, as well as contributions from all the other species that are considered synonymous to the species. This would explain the presence of CAN contamination in the hybrids, while also justifying the difference in accuracy of recognition of CAN versus NIL samples.

Similarly, further investigation of the relationship between *O. niloticus* and *O. aureus* revealed that the significant incidence of AUR contribution in the NIL samples as well as the breeding hybrids was not an isolated occurrence of this project. Different studies^17,27^ observed that the two species are likely to share a common ancestral mitochondrial haplotype: the presence of some degree of hybridisation between these two species would explain why, even though AUR is genetically distant from all other populations, the AUR species has such a significant presence in the ancestry assignment of NIL and NIL-derived samples, while also being absent from all other species.

Regarding the quality of the breeding hybrids, the analysis of their ancestry showed that, on average, more than 90% of their genome derived from *O. niloticus*, with only minor contributions from other species. These results showed that the pipeline is capable of confidently recognising the ancestry of admixed individuals, as the hybrids analysed were indeed descendants of *O. niloticus*. Fish from the GIFT breeding programme have already been shown as having a small contribution from *O. mossambisus* genome and *O. aureus*^28–30^. The actual extent depends on the methodology used, but it ranges from 1 to 7% for *O. mossambisus* and 0.5% to 3% for *O. aureus*. In this study, which used a genome wide approach, we identified the contribution to be on average 3% and 2% for *O. mossambisus* and *O. aureus* respectively.

Hybridisation is common and affects original phenotypes in most of the areas. However, it lacks the suitable method to identify the individual derived from hybrid and introgression in a simple and inexpensive way. We report, the new pipeline which could be used in further to evaluate the most the marker-based studies without further expensive experimental sampling or sequencing. This methodology based on ddRAD SNP markers has shown itself capable of identifying the contribution of multiple ancestral populations to the genome of admixed individuals, both from a local and global perspective. If provided with a large body of fully genotyped populations of known origin, the results produced by this pipeline would contribute to a more informed breeding process for the creation and maintenance of tilapia variants.

## Methods

### Biological materials

Fin samples were collected from a total of 71 individuals from four breeding programmes located in Mexico: Colima (BRE-C), Morelos (BRE-M) and Veracruz (BRE-V) broodstock; descendants from the Institute of Aquaculture fish, over 15 years ago. BRE-L, YY fish were obtained from a stock originating in Costa Rica. An additional 6 *O. mossambicus* (3 MOS-A and 3 MOS-Z) and 16 *O. niloticus* (NIL) reference samples were included. Samples were stored in 95% ethanol at − 20 °C until required. Details of the samples and origins are listed in Table 1. Attempts were made to balance the sex ratios (Supplementary Table S1 online) in order to minimise any potential bias due to sex-specific regions of the genome.

### Genomic DNA extraction

Purified DNA was extracted by a modified salt precipitation method^30^. Small pieces of fin tissue were digested in 300 μL SSTNE lysis solution (0.3 M NaCl, 50 mM Tris base, 0.2 mM EDTA pH 8.0, 0.2 mM EGTA, 0.5 mM spermidine, 0.25 mM spermine and 0.1% SDS) containing 1.5 μL Proteinase K (10 mg/mL) at 55 °C overnight. Lysed samples were treated with 5 μL RNaseA (2 mg/mL) at 37 °C for 1 h and the supernatant centrifuged twice at 21,000×*g* after precipitation with 180 μL 5 M NaCl on ice. The resulting DNA was precipitated in an equal volume of isopropanol, washed twice in 70% ethanol and dissolved in TE buffer (10 mM Tris, 1 mM EDTA pH 8.0) until DNA quantification. The quantity and quality of DNA were assessed by measurement on a Nanodrop spectrophotometer (Labtech International Ltd, UK) and by agarose gel electrophoresis. Standardised dilutions of 8 ng/μL DNA for each sample were prepared in 5 mM Tris buffer pH 8.0 according to fluorimetry values.

### Double Digest RAD library preparation and sequencing

Two libraries were constructed (Supplementary Table S1 online) following the ddRAD library preparation protocol with slight modifications^11^. Briefly, for each library, individual DNA samples (36 ng–4.5 μL) were simultaneously digested with two high fidelity restriction enzymes (New England Biolabs, NEB, UK): *Sbf*I (CCTGCA|GG recognition site), and *Sph*I (GCATG|C recognition site). Digestions were incubated for 90 min at 37 °C, using 0.72 U of each enzyme in 1× CutSmart Buffer (NEB) and in a 9 μL reaction volume. The reactions were then cooled to 22 °C, 4.5 μL of a pre-made barcode/adaptor mix was added to each digested DNA sample and incubated at 22 °C for 10 min. The adaptor mix included individual-specific barcoded combinations of P1 (*Sbf*I-compatible) and P2 (*Sph*I-compatible) adaptors at 6 nM and 72 nM concentrations respectively, in 1× reaction buffer 2 (NEB). The adaptors included an inline five- or seven-base barcode for sample identification. Ligation was performed over 2.5 h at 22 °C by addition of a further 4.5 μL of a ligation mix including 4 mM rATP (Promega, UK), and 2000 cohesive-end units of T4 ligase (NEB) per μg DNA in 1× CutSmart buffer. Samples for each library were combined into a single pool. The pooled libraries were column-purified (MinElute PCR Purification Kit, Qiagen, UK), and eluted in 60 μL EB buffer (Qiagen, UK). Size-selection of fragments, ranging from 320 to 590 bp, was performed by agarose gel separation. Following gel purification (MinElute Gel Extraction Kit, Qiagen, UK), the eluted size-selected template DNA (65 μL in EB buffer) was PCR amplified (11–12 cycles PCR dependent on library; 32 separate 12.5 μL reactions, each with 1.25 μL template DNA) using a high fidelity Taq polymerase (Q5 Hot Start High-Fidelity DNA Polymerase, NEB). The PCR reactions were combined (400 μL total), and column-purified (MinElute PCR Purification Kit). The c. 50 μL eluate, in EB buffer, was then subjected to a further size-selection clean up using an equal volume of AMPure magnetic beads (Perkin-Elmer), to maximise removal of small fragments (less than c. 200 bp). Each final library was eluted in 15 μL EB buffer, QUBIT quantified and diluted to 10 nM stocks and sequenced in house on a separate Illumina MiSeq run (v2 chemistry, 300 cycle kit, 150 base paired-end reads).

### Data origins

A total of 10 different species^11,12^, along with individuals sourced from breeding programmes, were used to produce ddRAD markers^31^ following the same protocol: restriction enzymes set (*Sbf*I and *Sph*I), size selection (320 bp to 590 bp) and comparable sequencing platforms (150 nucleotide paired-ends), rendering their results compatible. Efforts were made to use populations with known histories, an absence of hybridisation, and from multiple locations (Table 1). The *O. niloticus* samples consisted of three sub-species (*O. niloticus sensu stricto* and *O. n. filoa* and *O. n. cancellatus*); *O. aureus*, *O. mossambicus* and *Tilapia zillii* (Gervais: reclassification as *Coptodon zillii* proposed by Dunz and Schliewen^32^) comprised samples from two locations each, while *O. karongae* (Trewavas), *O. urolepis hornorum* (Norman), *O. andersonii*, *O. macrochir*, *Sarotherodon galilaeus* (Linnaeus) and *S. melanotheron* consisted of samples from one location each. As far as could be ascertained, each originated from a single wild population (in some cases then maintained and bred in captivity). Additionally, a ddRAD dataset from the popular Genetically Improved Farmed Tilapia (GIFT) breeding programme^13^ and samples from four breeding programmes in Mexico were assessed (Table 1).

### Dataset preparation

Reads of low quality (i.e., with an average quality score less than 20), lacking the restriction site or having ambiguous barcodes were discarded during the samples demultiplexing stage. Retained reads were aligned against the genomic assembly of the tilapia species *O. niloticus* (NCBI Assembly accession GCA_001858045.3) using bwa^33^ and assembled using Stack^34^. Markers produced through ddRAD sequencing were collected from the 275 samples. All loci that were common to at least 4 populations and at least 50% of their individuals, a minor allele frequency over 0.05 and not deviating from the expected Mendelian segregation (*P* > 0.01) were retained, as the missing data could be inferred by imputation.

### Ancestry inference

BEAGLE^19^ was used for the phasing of genotypes. BEAGLE performs multiple phasing iterations per SNP. After the phasing was carried out and the model was fit, the data were analysed again to obtain new estimates that allowed a better refit of the model. RFMix^23^ was used for LAI. To optimise the inference accuracy using only the 155 references samples from 10 species, the number of phasing iterations, number of expectation-maximisation (EM) iterations, and the chromosomal window size were varied, and their results were compared. The combination of parameters that produced the least amount of fragmentation in theoretically pure individuals was chosen as most suitable.

### Multidimensional scaling analysis

R v3.5.2^35^ was used to carry out Multidimensional Scaling Analysis on the dataset using the package R/SNPRelate v1.16.0^36^ to calculate the Identity-By-State (IBS) proportion for each sample.

### Digital chromosome painting

Inferred local ancestry data, produced by RFMix, were visualised using R for the distribution of the local probabilities, and a dedicated script rendered the final distribution as a painted karyotype for each sample. Full scripts and pipelines are available on GitHub at https://github.com/pseudogene/fish_pedigree.

### Ethical approval

Animal handling and collection was conducted under the UK Home Office guidelines and regulations [Samples MOZ-A/Z and NIL] and the Michoacán de Ocampo authority (Mexico) guidelines and regulations [Samples from the Breeding programme; BRE-C/L/M/V]. The ethical approval for the study was obtained from the University of Stirling (UK) Ethical committee. The data analytics and bioinformatics were assessed by the Institute of Aquaculture Ethical Review Committee and passed the University of Stirling Ethical Review Process.

## Supplementary information

Supplementary Information 1.

Supplementary Information 2.

Supplementary Information 3.

## Data Availability

The raw sequencing reads generated for this article are available from the EBI/ENA, accession Numbers ERR4170942 and ERR4171069, project PRJEB38387.

## Code availability

The versions, settings and parameters of the software used in this work are as follows: (1) **process_radtags.pl**: Stack version 2.3, parameters: -E phred33 -filter_illumina -s 20 -c -q -t 135 -inline_inline -renz_1 sbfI -renz_2 sphI; (2) **bwa**: version 0.7.17, default parameters; (3) **ref_map.pl**: Stack version 2.3, parameters: -unpaired; (4) **populations**: Stack version 2.3, parameters: -write-single-snp -p 4 -r 50 -R 50 -min-maf 0.05 -hwe -vcf; (5) **BEAGLE**: version 5.1, parameters: java -Xmx30688m -jar beagle.21Sep19.ec3.jar imp-nsteps=50 iterations=1000 window=0.7 overlap=0.07; (6) **RFMix**: version 2.03, parameters: -e 50.

## References

[1] Huson HJ (2012). Breed-specific ancestry studies and genome-wide association analysis highlight an association between the myh9 gene and heat tolerance in alaskan sprint racing sled dogs. Mamm. Genome.

[2] Henn BM (2012). Genomic ancestry of North Africans supports back-to-africa migrations. PLoS Genet..

[3] Martin AR (2017). Human demographic history impacts genetic risk prediction across diverse populations. Am. J. Hum. Genet..

[4] Tang H, Coram M, Wang P, Zhu X, Risch N (2006). Reconstructing genetic ancestry blocks in admixed individuals. Am. J. Hum. Genet..

[5] Geza E (2018). A comprehensive survey of models for dissecting local ancestry deconvolution in human genome. Brief. Bioinform..

[6] Corbett-Detig R, Nielsen R (2017). A hidden markov model approach for simultaneously estimating local ancestry and admixture time using next generation sequence data in samples of arbitrary ploidy. PLoS Genet..

[7] Choi BH (2017). Genome-wide analysis of the diversity and ancestry of korean dogs. PLoS ONE.

[8] Novembre J (2008). Genes mirror geography within Europe. Nature.

[9] Clarke L (2012). The 1000 genomes project: data management and community access. Nat. Methods.

[10] Gupta MV, Acosta BO (2004). From drawing board to dining table: the success story of the gift project. NAGA, WorldFish Center Q..

[11] Syaifudin M (2019). Species-specific marker discovery in Tilapia. Sci. Rep..

[12] Syaifudin, M., McAndrew, B. J. & Penman, D. J. *Species-Specific DNA Markers for Improving the Genetic Management of Tilapia*. Ph. D. thesis, University of Stirling (2015).

[13] Taslima K (2020). Sex determination in the gift strain of Tilapia is controlled by a locus in linkage group 23. BMC Genet..

[14] Miller MR, Dunham JP, Amores A, Cresko WA, Johnson EA (2007). Rapid and cost-effective polymorphism identification and genotyping using restriction site associated dna (rad) marker. Genome Res..

[15] Baird NA (2008). Rapid snp discovery and genetic mapping using sequenced rad markers. PLoS ONE.

[16] Jolliffe IT, Cadima J (2016). Principal component analysis: a review and recent developments. Philos. Trans. R. Soc. A Math. Phys. Eng. Sci..

[17] Bezault E (2011). Spatial and temporal variation in population genetic structure of wild Nile tilapia (*Oreochromis niloticus*) across Africa. BMC Genet..

[18] Seyoum S, Kornfield I (1992). Taxonomic notes on the Oreochromis niloticus subspecies-complex (pisces: Cichlidae), with a description of a new subspecies. Can. J. Zool..

[19] Browning SR, Browning BL (2007). Rapid and accurate haplotype phasing and missing-data inference for wole-genome association studies by use of localized haplotype clustering. Am. J. Hum. Genet..

[20] Padhukasahasram B (2014). Inferring ancestry from population genomic data and its applications. Front. Genet..

[21] Moreno-Estrada A (2013). Reconstructing the population genetic history of the caribbean. PLoS Genet..

[22] Tewhey R, Bansal V, Torkamani A, Topol EJ, Schork NJ (2011). The importance of phase information for human genomics. Nat. Rev. Genet..

[23] Maples BK, Gravel S, Kenny EE, Bustamante CD (2013). Rfmix: a discriminative modeling approach for rapid and robust local-ancestry inference. Am. J. Hum. Genet..

[24] Gravel S (2012). Population genetics models of local ancestry. Genetics.

[25] Lemarié G, Baroiller JF, Clota F, Lazard J, Dosdat A (2004). A simple test to estimate the salinity resistance of fish with specific application to *O. niloticus* and *S. melanotheron*. Aquaculture.

[26] Nichols, J. T. A new wrasse and two new cichlids from Northeast Africa. *Am. Museum Novitat* **65**, 1–4 (1923).

[27] Agnèse JF, Adépo-Gourène B, Abban EK, Fermon Y (1997). Genetic differentiation among natural populations of the nile tilapia *Oreochromis niloticus* (teleostei, cichlidae). Heredity.

[28] Van Bers NEM, Crooijmans RPMA, Groenen MAM, Dibbits BW, Komen J (2012). SNP marker detection and genotyping in tilapia. Mol. Ecol. Resour..

[29] Hong Xia J (2015). Signatures of selection in tilapia revealed by whole genome resequencing. Sci. Rep..

[30] Bartie KL (2020). Species composition in the molobicus hybrid Tilapia strain. Aquaculture.

[31] Peterson BK, Weber JN, Kay EH, Fisher HS, Hoekstra HE (2012). Double digest radseq: an inexpensive method for de novo snp discovery and genotyping in model and non-model species. PLoS ONE.

[32] Dunz AR, Schliewen UK (2013). Molecular phylogeny and revised classification of the haplotilapiine cichlid fishes formerly referred to as Tilapia. Mol. Phylogenet. Evol..

[33] Li H, Durbin R (2009). Fast and accurate short read alignment with burrows-wheeler transform. Bioinformatics.

[34] Catchen JM, Amores A, Hohenlohe P, Cresko W, Postlethwait JH (2011). Stacks: building and genotyping loci de novo from short-read sequences. G3 Genes Genomes Genet..

[35] R Core Team. *R: A Language and Environment for Statistical Computing*. R Foundation for Statistical Computing, Vienna, Austria (2019).

[36] Zheng X (2012). A high-performance computing toolset for relatedness and principal component analysis of SNP data. Bioinformatics.

