# Supplementary material for: Local ancestry inference provides insight into Tilapia breeding programmes
Source: Sci Rep. 2020 Oct 29;10:18613. doi: 10.1038/s41598-020-75744-9 (PMC7596482; doi:10.1038/s41598-020-75744-9)
Supplement: Supplementary file 1 — Supplementary Information 1. [file 41598_2020_75744_MOESM1_ESM.pdf]

Local ancestry inference provides insight into Tilapia breeding programmes

Alex Avallone[1], Kerry L Bartie[1], Sarah-Louise C Selly[1], Khanam Taslima[1,2], Antonio Campos Mendoza[3], and Michaël Bekaert[1,*]

[1]Institute of Aquaculture, Faculty of Natural Sciences, University of Stirling, Stirling, FK9 4LA, Scotland, United-Kingdom
[2]Department of Fisheries Biology and Genetics, Bangladesh Agricultural University, Mymensingh, 2202, Bangladesh
[3]Faculty of Biology, Universidad Michoacana de San Nicolás de Hidalgo, Morelia, Michoacán, 58040, Mexico
[*]michael.bekaert@stir.ac.uk

Supplementary Information

- **Table S1**. Details of the sequenced library.
- **Table S2**. Details of the samples.
- **Data S3**. Phased haplotypes of the 275 samples and 19,041 markers. Each marker is located on the GCA_001858045.3 assembly (Variant Call Format - VCF).

Supplementary Information — Table S1

Table S1. Details of the sequenced library.

SampleID	Population	Reads	SampleREF
AND_1	AND	232758	Md-91
AND_2	AND	563288	Md-92
AND_3	AND	675174	Md-93
AND_4	AND	499538	Md-94
AND_5	AND	656560	Md-98
AND_6	AND	77544	Md-100
AUR-I_1	AUR-I	1192988	MM-247
AUR-I_2	AUR-I	1446800	Md-249
AUR-I_3	AUR-I	771570	Md-256
AUR-I_4	AUR-I	1011382	Md-257
AUR-I_5	AUR-I	1459244	Md-259
AUR-I_6	AUR-I	1431672	Md-260
AUR-I_7	AUR-I	1336868	Md-262
AUR-I_8	AUR-I	1718554	Md-263
AUR-I_9	AUR-I	1419794	Md-265
AUR-I_10	AUR-I	1194498	Md-266
AUR-E_1	AUR-E	1521692	Md-21
AUR-E_2	AUR-E	1310644	Md-22
AUR-E_3	AUR-E	972380	Md-23
AUR-E_4	AUR-E	2402842	Md-26
AUR-E_5	AUR-E	836666	Md-66
KAR_1	KAR	1114616	Md-29
KAR_2	KAR	875448	Md-30
KAR_3	KAR	1952048	Md-31
KAR_4	KAR	1077908	Md-32
MAC_1	MAC	251368	Md-113

SampleID	Population	Reads	SampleREF
MAC_2	MAC	931152	Md-115
MAC_3	MAC	231874	Md-116
MAC_4	MAC	303394	Md-117
MOS-A_1	MOS-A	829562	Md-269
MOS-A_2	MOS-A	540828	Md-270
MOS-A_3	MOS-A	627674	Md-271
MOS-A_4	MOS-A	317922	Md-274
MOS-A_5	MOS-A	772408	Md-276
MOS-A_6	MOS-A	266204	Md-280
MOS-A_7	MOS-A	356380	Md-281
MOS-A_8	MOS-A	460402	Md-283
MOS-A_9	MOS-A	220052	Md-284
MOS-A_10	MOS-A	371932	Md-288
MOS-A_11	MOS-A	819500	MoS270
MOS-A_12	MOS-A	844518	MoS271
MOS-A_13	MOS-A	650532	MoS272
MOS-Z_1	MOS-Z	1612312	Md-12
MOS-Z_2	MOS-Z	990964	Md-14
MOS-Z_3	MOS-Z	1349408	Md-15
MOS-Z_4	MOS-Z	1017114	Md-16
MOS-Z_5	MOS-Z	848560	Md-17
MOS-Z_6	MOS-Z	801202	Md-33
MOS-Z_7	MOS-Z	919344	MoS043
MOS-Z_8	MOS-Z	1003260	MoS045
MOS-Z_9	MOS-Z	1014570	MoS046
CAN_1	CAN	234006	Md-174
CAN_2	CAN	287204	Md-175
CAN_3	CAN	243358	Md-176
CAN_4	CAN	429310	Md-177
CAN_5	CAN	318984	Md-178
CAN_6	CAN	287244	Md-179
CAN_7	CAN	307572	Md-180
CAN_8	CAN	266030	Md-182
CAN_9	CAN	394908	Md-184
CAN_10	CAN	428914	Md-186
CAN_11	CAN	378424	Md-188
CAN_12	CAN	854152	Md-189
CAN_13	CAN	439406	Md-190
CAN_14	CAN	552660	Md-191
CAN_15	CAN	475644	Md-194
CAN_16	CAN	438622	Md-195
CAN_17	CAN	442790	Md-196
CAN_18	CAN	353382	Md-197
CAN_19	CAN	292802	Md-198
CAN_20	CAN	245206	Md-200
CAN_21	CAN	330730	Md-201
CAN_22	CAN	467128	Md-202
CAN_23	CAN	328960	Md-203
CAN_24	CAN	341820	Md-204
CAN_25	CAN	329072	Md-210
CAN_26	CAN	1477568	Md-215
CAN_27	CAN	2620938	Md-221
CAN_28	CAN	1375230	Md-222
CAN_29	CAN	1272604	Md-223

SampleID	Population	Reads	SampleREF
CAN_30	CAN	877824	Md-224
CAN_31	CAN	884208	Md-225
CAN_32	CAN	555372	Md-226
CAN_33	CAN	783578	Md-228
BRE-C_1	BRE-C	643808	Col340
BRE-C_2	BRE-C	252650	Col341
BRE-C_3	BRE-C	487904	Col342
BRE-C_4	BRE-C	774416	Col343
BRE-C_5	BRE-C	864572	Col345
BRE-C_6	BRE-C	471104	Col346
BRE-C_7	BRE-C	693258	Col348
BRE-C_8	BRE-C	404084	Col349
BRE-C_9	BRE-C	1212212	Col350
BRE-C_10	BRE-C	444746	Col351
BRE-C_11	BRE-C	135864	Col352
BRE-C_12	BRE-C	451818	Col353
BRE-C_13	BRE-C	303074	Col354
BRE-C_14	BRE-C	238410	Col355
BRE-C_15	BRE-C	397950	Col356
BRE-C_16	BRE-C	594112	Col357
BRE-C_17	BRE-C	177724	Col358
BRE-M_1	BRE-M	644796	Mor162
BRE-M_2	BRE-M	882650	Mor163
BRE-M_3	BRE-M	546612	Mor164
BRE-M_4	BRE-M	1022834	Mor165
BRE-M_5	BRE-M	917340	Mor167
BRE-M_6	BRE-M	720088	Mor169
BRE-M_7	BRE-M	821382	Mor170
BRE-M_8	BRE-M	524282	Mor171
BRE-M_9	BRE-M	695726	Mor172
BRE-M_10	BRE-M	902812	Mor283
BRE-M_11	BRE-M	882960	Mor284
BRE-M_12	BRE-M	958198	Mor285
BRE-M_13	BRE-M	948006	Mor286
BRE-M_14	BRE-M	804972	Mor287
BRE-M_15	BRE-M	599596	Mor288
BRE-M_16	BRE-M	820896	Mor289
BRE-M_17	BRE-M	960794	Mor290
BRE-M_18	BRE-M	850150	Mor292
BRE-V_1	BRE-V	360054	Ver301
BRE-V_2	BRE-V	584586	Ver302
BRE-V_3	BRE-V	344196	Ver303
BRE-V_4	BRE-V	576690	Ver305
BRE-V_5	BRE-V	320814	Ver306
BRE-V_6	BRE-V	248066	Ver310
BRE-V_7	BRE-V	1067456	Ver312
BRE-V_8	BRE-V	366612	Ver313
BRE-V_9	BRE-V	218646	Ver315
BRE-V_10	BRE-V	863510	Ver316
BRE-V_11	BRE-V	723176	Ver317
BRE-V_12	BRE-V	418892	Ver318
BRE-V_13	BRE-V	341066	Ver319
BRE-V_14	BRE-V	87506	Ver320
BRE-V_15	BRE-V	207690	Ver321

SampleID	Population	Reads	SampleREF
BRE-V_16	BRE-V	189380	Ver322
BRE-V_17	BRE-V	596170	Ver323
BRE-V_18	BRE-V	341418	Ver324
GIFT_1	GIFT	223978	025Dam
GIFT_2	GIFT	272684	025Sir
GIFT_3	GIFT	286942	027Dam
GIFT_4	GIFT	439492	027Sir
GIFT_5	GIFT	360802	031Dam
GIFT_6	GIFT	219238	031Sir
GIFT_7	GIFT	322458	033Dam
GIFT_8	GIFT	496994	037Dam
GIFT_9	GIFT	373794	037Sir
GIFT_10	GIFT	288112	040Dam
GIFT_11	GIFT	228716	040Sir
GIFT_12	GIFT	442930	044Dam
GIFT_13	GIFT	456032	044Sir
GIFT_14	GIFT	280568	048Sir
GIFT_15	GIFT	348476	050Dam
GIFT_16	GIFT	236190	050Sir
GIFT_17	GIFT	362078	051Dam
GIFT_18	GIFT	359204	051Sir
GIFT_19	GIFT	389620	052Dam
GIFT_20	GIFT	352392	052Sir
GIFT_21	GIFT	406408	053Dam
GIFT_22	GIFT	457066	053Sir
GIFT_23	GIFT	415244	055Sir
GIFT_24	GIFT	422424	056Dam
GIFT_25	GIFT	367192	056Sir
GIFT_26	GIFT	344336	058Dam
GIFT_27	GIFT	337504	058Sir
GIFT_28	GIFT	283902	059Dam
GIFT_29	GIFT	356178	080Sir
GIFT_30	GIFT	352882	087Dam
GIFT_31	GIFT	517836	087Sir
GIFT_32	GIFT	407966	092Sir
GIFT_33	GIFT	980788	097Dam
GIFT_34	GIFT	793022	097Sir
GIFT_35	GIFT	658254	099Dam
GIFT_36	GIFT	606234	099Sir
GIFT_37	GIFT	723898	09Dam
GIFT_38	GIFT	833146	09Sire
GIFT_39	GIFT	461390	101Dam
GIFT_40	GIFT	246598	101Sir
GIFT_41	GIFT	384294	105Dam
GIFT_42	GIFT	246246	105Sir
GIFT_43	GIFT	882758	12Dam
GIFT_44	GIFT	1001384	12Sire
GIFT_45	GIFT	832238	13Dam
GIFT_46	GIFT	1109000	13Sire
GIFT_47	GIFT	605276	15Dam
GIFT_48	GIFT	654698	15Sire
GIFT_49	GIFT	853342	23Dam
GIFT_50	GIFT	733464	23Sire
BRE-L_1	BRE-L	676390	Myy001

SampleID	Population	Reads	SampleREF
BRE-L_2	BRE-L	457502	Myy006
BRE-L_3	BRE-L	469624	Myy007
BRE-L_4	BRE-L	670652	Myy009
BRE-L_5	BRE-L	714076	Myy010
BRE-L_6	BRE-L	818016	Myy011
BRE-L_7	BRE-L	768228	Myy012
BRE-L_8	BRE-L	773874	Myy013
BRE-L_9	BRE-L	806734	Myy014
BRE-L_10	BRE-L	571862	Myy015
BRE-L_11	BRE-L	736472	Myy016
BRE-L_12	BRE-L	794586	Myy017
BRE-L_13	BRE-L	847568	Myy018
BRE-L_14	BRE-L	572726	Myy019
BRE-L_15	BRE-L	830514	Myy020
BRE-L_16	BRE-L	568470	Myy022
BRE-L_17	BRE-L	723154	Myy023
BRE-L_18	BRE-L	414832	Myy024
NIL_1	NIL	1176840	Md-134
NIL_2	NIL	777810	Md-136
NIL_3	NIL	316210	Md-137
NIL_4	NIL	497834	Md-138
NIL_5	NIL	2031356	Md-140
NIL_6	NIL	449728	Md-142
NIL_7	NIL	543328	Md-143
NIL_8	NIL	398318	Md-144
NIL_9	NIL	413474	Md-145
NIL_10	NIL	339772	Md-149
NIL_11	NIL	544544	Md-150
NIL_12	NIL	507768	Md-152
NIL_13	NIL	543338	Md-154
NIL_14	NIL	432792	Md-156
NIL_15	NIL	225832	Md-157
NIL_16	NIL	245488	Md-158
NIL_17	NIL	2114242	Md-160
NIL_18	NIL	312468	Md-162
NIL_19	NIL	309288	Md-163
NIL_20	NIL	200390	Md-164
NIL_21	NIL	229904	Md-165
NIL_22	NIL	280092	Md-170
NIL_23	NIL	525148	Md-172
NIL_24	NIL	291242	Md-173
NIL_25	NIL	1046082	Md-08
NIL_26	NIL	1246958	Md-09
NIL_27	NIL	1377062	Md-10
NIL_28	NIL	1109838	Md-11
NIL_29	NIL	1172038	Md-498
NIL_30	NIL	1338556	Md-499
NIL_31	NIL	516168	Sti025
NIL_32	NIL	610644	Sti028
NIL_33	NIL	624254	Sti031
NIL_34	NIL	299926	Sti034
NIL_35	NIL	387360	Sti035
NIL_36	NIL	554416	Sti037
NIL_37	NIL	494038	Sti039

SampleID	Population	Reads	SampleREF
NIL_38	NIL	495118	Sti040
NIL_39	NIL	232130	Sti042
NIL_40	NIL	733640	Sti043
NIL_41	NIL	770792	Sti044
NIL_42	NIL	505282	Sti045
NIL_43	NIL	432382	Sti046
NIL_44	NIL	313778	Sti048
NIL_45	NIL	473164	Sti050
NIL_46	NIL	409374	Sti051
HOR_1	HOR	570664	M69
HOR_2	HOR	718066	M70
HOR_3	HOR	737788	M75
HOR_4	HOR	802414	M77
HOR_5	HOR	638506	MM-78
GAL_1	GAL	673834	MM-294
GAL_2	GAL	529064	MM-298
GAL_3	GAL	915488	MM-301b
GAL_4	GAL	981768	MM-302
GAL_5	GAL	660892	Md-309
MEL_1	MEL	233014	Md-230
MEL_2	MEL	240600	Md-231
MEL_3	MEL	3203984	Md-234
MEL_4	MEL	311392	Md-235
ZIL_1	ZIL	1471698	Md-237a
ZIL_2	ZIL	237680	Md-242
ZIL_3	ZIL	250400	Md-246a
ZIL_4	ZIL	274682	Md-246b
ZIL_5	ZIL	671644	MM-246c
ZIL_6	ZIL	856184	Md-35
ZIL_7	ZIL	1509366	Md-37
ZIL_8	ZIL	1012510	Md-38
ZIL_9	ZIL	1065834	Md-39
ZIL_10	ZIL	1453648	Md-40

Supplementary Information — Table S2

Table S2. Details of the samples.

SampleID	Reads	Gender	Library	Barcode1	Barcode2	Collection_ID	SampleREF
MOS-A_11	819500	F	Library 2	ACGTA	CTGGT	MoS270	MM_270
MOS-A_12	844518	F	Library 2	CAGTCAC	GTCAAGT	MoS271	MM_271
MOS-A_13	650532	F	Library 2	AGAGT	GAAGC	MoS272	MM_272
MOS-Z_7	919344	M	Library 2	GCTAACA	ATACGGT	MoS043	MM_43
MOS-Z_8	1003260	F	Library 2	CGATA	CGATC	MoS045	MM_45
MOS-Z_9	1014570	F	Library 2	CTAGGAC	CATCTGT	MoS046	MM_46
BRE-C_1	643808	F	Library 2	GTACACA	GCATA	Col340	MX.LINK.340
BRE-C_2	252650	F	Library 2	GCATT	GAGATGT	Col341	MX.LINK.341
BRE-C_3	487904	F	Library 1	CATGA	ATACGGT	Col342	MX.LINK.342
BRE-C_4	774416	F	Library 1	CACAGAC	TAGCA	Col343	MX.LINK.343
BRE-C_5	864572	F	Library 1	ATCGA	AGCTGTC	Col345	MX.LINK.345
BRE-C_6	471104	F	Library 2	CTCTTCA	CGATC	Col346	MX.LINK.346
BRE-C_7	693258	F	Library 1	ACTGCAC	AGTCA	Col348	MX.LINK.348

SampleID	Reads	Gender	Library	Barcode1	Barcode2	Collection_ID	SampleREF
BRE-C_8	404084	F	Library 1	TCGAG	TACGTGT	Col349	MX.LINK.349
BRE-C_9	1212212	F	Library 2	CGATA	CATCTGT	Col350	MX.LINK.350
BRE-C_10	444746	M	Library 1	TCTCTCA	GCATA	Col351	MX.LINK.351
BRE-C_11	135864	M	Library 1	GTCAC	GAGATGT	Col352	MX.LINK.352
BRE-C_12	451818	M	Library 1	GTACACA	CGATC	Col353	MX.LINK.353
BRE-C_13	303074	M	Library 2	CTAGGAC	CTGGT	Col354	MX.LINK.354
BRE-C_14	238410	M	Library 2	ACGTA	GTCAAGT	Col355	MX.LINK.355
BRE-C_15	397950	M	Library 2	CAGTCAC	GAAGC	Col356	MX.LINK.356
BRE-C_16	594112	M	Library 1	GCATT	CATCTGT	Col357	MX.LINK.357
BRE-C_17	177724	M	Library 1	CTCTTCA	CTGGT	Col358	MX.LINK.358
BRE-M_1	644796	M	Library 1	AGGACAC	GAGATGT	Mor162	MX.LINK.162
BRE-M_2	882650	M	Library 1	TCAGA	CATCTGT	Mor163	MX.LINK.163
BRE-M_3	546612	M	Library 1	TGCAACA	CTGGT	Mor164	MX.LINK.164
BRE-M_4	1022834	M	Library 2	ACTGCAC	TAGCA	Mor165	MX.LINK.165
BRE-M_5	917340	M	Library 1	GATCG	GTCAAGT	Mor167	MX.LINK.167
BRE-M_6	720088	M	Library 2	TCGAG	AGCTGTC	Mor169	MX.LINK.169
BRE-M_7	821382	M	Library 1	CGTATCA	GAAGC	Mor170	MX.LINK.170
BRE-M_8	524282	M	Library 2	TCTCTCA	AGTCA	Mor171	MX.LINK.171
BRE-M_9	695726	M	Library 2	GTCAC	TACGTGT	Mor172	MX.LINK.172
BRE-M_10	902812	F	Library 2	CGTATCA	CTGGT	Mor283	MX.LINK.283
BRE-M_11	882960	F	Library 1	AGAGT	TAGCA	Mor284	MX.LINK.284
BRE-M_12	958198	F	Library 1	GCTAACA	AGCTGTC	Mor285	MX.LINK.285
BRE-M_13	948006	F	Library 1	ATGCT	AGTCA	Mor286	MX.LINK.286
BRE-M_14	804972	F	Library 1	ACACGAG	TACGTGT	Mor287	MX.LINK.287
BRE-M_15	599596	F	Library 1	GACTA	GCATA	Mor288	MX.LINK.288
BRE-M_16	820896	F	Library 2	CATGA	GTCAAGT	Mor289	MX.LINK.289
BRE-M_17	960794	F	Library 2	CACAGAC	GAAGC	Mor290	MX.LINK.290
BRE-M_18	850150	F	Library 2	ATCGA	ATACGGT	Mor292	MX.LINK.292
BRE-V_1	360054	M	Library 1	CTAGGAC	GAAGC	Ver301	MX.LINK.301
BRE-V_2	584586	M	Library 2	AGAGT	ATACGGT	Ver302	MX.LINK.302
BRE-V_3	344196	M	Library 1	ACGTA	ATACGGT	Ver303	MX.LINK.303
BRE-V_4	576690	M	Library 1	CAGTCAC	TAGCA	Ver305	MX.LINK.305
BRE-V_5	320814	M	Library 1	AGAGT	AGCTGTC	Ver306	MX.LINK.306
BRE-V_6	248066	M	Library 1	GCTAACA	AGTCA	Ver310	MX.LINK.310
BRE-V_7	1067456	M	Library 2	GCTAACA	TAGCA	Ver312	MX.LINK.312
BRE-V_8	366612	M	Library 1	ATGCT	TACGTGT	Ver313	MX.LINK.313
BRE-V_9	218646	M	Library 1	ACACGAG	GCATA	Ver315	MX.LINK.315
BRE-V_10	863510	M	Library 2	ATGCT	AGCTGTC	Ver316	MX.LINK.316
BRE-V_11	723176	M	Library 2	ACACGAG	AGTCA	Ver317	MX.LINK.317
BRE-V_12	418892	M	Library 2	GACTA	TACGTGT	Ver318	MX.LINK.318
BRE-V_13	341066	M	Library 2	AGGACAC	GCATA	Ver319	MX.LINK.319
BRE-V_14	87506	M	Library 1	GACTA	GAGATGT	Ver320	MX.LINK.320
BRE-V_15	207690	M	Library 2	TCAGA	CGATC	Ver321	MX.LINK.321
BRE-V_16	189380	M	Library 1	AGGACAC	CGATC	Ver322	MX.LINK.322
BRE-V_17	596170	F	Library 1	CGATA	GTCAAGT	Ver323	MX.LINK.323
BRE-V_18	341418	M	Library 2	TGCAACA	CATCTGT	Ver324	MX.LINK.324
BRE-L_1	676390	M	Library 1	GACTA	TACGTGT	Myy001	1
BRE-L_2	457502	M	Library 2	TCAGA	GCATA	Myy006	18
BRE-L_3	469624	M	Library 2	TGCAACA	GAGATGT	Myy007	11
BRE-L_4	670652	M	Library 1	AGGACAC	GCATA	Myy009	13
BRE-L_5	714076	M	Library 1	TCAGA	CGATC	Myy010	15
BRE-L_6	818016	M	Library 1	TGCAACA	CATCTGT	Myy011	16
BRE-L_7	768228	M	Library 2	GATCG	CGATC	Myy012	19
BRE-L_8	773874	M	Library 1	GATCG	CTGGT	Myy013	26

SampleID	Reads	Gender	Library	Barcode1	Barcode2	Collection_ID	SampleREF
BRE-L_9	806734	M	Library 2	CGTATCA	CATCTGT	Myy014	31
BRE-L_10	571862	M	Library 2	CATGA	CTGGT	Myy015	35
BRE-L_11	736472	M	Library 2	CACAGAC	GTCAAGT	Myy016	36
BRE-L_12	794586	M	Library 2	ATCGA	GAAGC	Myy017	38
BRE-L_13	847568	M	Library 1	CGTATCA	GTCAAGT	Myy018	39
BRE-L_14	572726	M	Library 2	ACTGCAC	ATACGGT	Myy019	48
BRE-L_15	830514	M	Library 1	CATGA	GAAGC	Myy020	50
BRE-L_16	568470	M	Library 1	CACAGAC	ATACGGT	Myy022	94
BRE-L_17	723154	M	Library 1	ATCGA	TAGCA	Myy023	102
BRE-L_18	414832	M	Library 1	ACTGCAC	AGCTGTC	Myy024	128
NIL_31	516168	F	Library 1	TCGAG	AGTCA	Sti025	51F
NIL_32	610644	M	Library 1	CTCTTCA	CATCTGT	Sti028	12F
NIL_33	624254	M	Library 1	CGATA	CTGGT	Sti031	64F
NIL_34	299926	M	Library 2	GTACACA	TACGTGT	Sti034	CE248
NIL_35	387360	F	Library 1	TCTCTCA	TACGTGT	Sti035	11CB1
NIL_36	554416	F	Library 1	GTCAC	GCATA	Sti037	9CB1
NIL_37	494038	F	Library 2	TCGAG	TAGCA	Sti039	17CB2
NIL_38	495118	M	Library 2	GCATT	GCATA	Sti040	KAR4
NIL_39	232130	F	Library 2	TCTCTCA	AGCTGTC	Sti042	KAR3
NIL_40	733640	M	Library 1	CTAGGAC	GTCAAGT	Sti043	KAR1
NIL_41	770792	M	Library 1	ACGTA	GAAGC	Sti044	KM804
NIL_42	505282	M	Library 1	CAGTCAC	ATACGGT	Sti045	KAR2
NIL_43	432382	F	Library 1	GTACACA	GAGATGT	Sti046	KAR9
NIL_44	313778	M	Library 2	CTCTTCA	GAGATGT	Sti048	KAR5
NIL_45	473164	F	Library 1	GCATT	CGATC	Sti050	14CB4
NIL_46	409374	F	Library 2	GTCAC	AGTCA	Sti051	0068D005E

Supplementary Information — Data S3

See external Variant Call Format (VCF) file.
