# Supplementary material for: Local ancestry inference provides insight into Tilapia breeding programmes
Source: Sci Rep. 2020 Oct 29;10:18613. doi: 10.1038/s41598-020-75744-9 (PMC7596482; doi:10.1038/s41598-020-75744-9)
Supplement: Supplementary file 2 — Supplementary Information 2. [file 41598_2020_75744_MOESM2_ESM.pdf]

Local ancestry inference provides insight into Tilapia breeding programmes

Alex Avallone[1], Kerry L Bartie[1], Sarah-Louise C Selly[1], Khanam Taslima[1,2], Antonio Campos Mendoza[3], and Michaël Bekaert[1,*]

[1]Institute of Aquaculture, Faculty of Natural Sciences, University of Stirling, Stirling, FK9 4LA, Scotland, United-Kingdom
[2]Department of Fisheries Biology and Genetics, Bangladesh Agricultural University, Mymensingh, 2202, Bangladesh
[3]Faculty of Biology, Universidad Michoacana de San Nicolás de Hidalgo, Morelia, Michoacán, 58040, Mexico
[*]michael.bekaert@stir.ac.uk

Supplementary Information

- **Table S1**. Details of the sequenced library.
- **Table S2**. Details of the samples.
- **Data S3**. Phased haplotypes of the 275 samples and 19,041 markers. Each marker is located on the GCA_001858045.3 assembly (Variant Call Format - VCF).

Supplementary Information — Data S3

See external Variant Call Format (VCF) file.
